# Microprocessor automation of a UV-visible monochromator

**DOI:** 10.1155/S1463924681000229

**Published:** 1981

**Authors:** Malcolm W. Warren, James P. Avery, Daniel W. Lovse, Howard V. Malmstadt

**Affiliations:** University of Illinois School of Chemical Sciences Department of Chemistry Urbana IL 61801 USA


### Microprocessor automation of a UV–visible monochromator

**Malcolm W. Warren, James P. Avery, Daniel W. Lovse, and Howard V. Malmstadt**
*University of Illinois, School of Chemical Sciences, Department of Chemistry, Urbana, IL 61801, USA.*

### Introduction

Isolation of selected wavelengths of light with high resolution grating monochromators is a common operation in spectrochemical methods of analysis. Commercial monochromators and monochromator controllers are often unable to control the monochromator's operating parameters with the resolution required in automated systems. If a monochromator is to be used in a completely automated system its controller should provide a number of important capabilities: initialisation of wavelength and slitwidth operation; calibration of the wavelength and slitwidth; the ability to select any wavelength available; the ability to set any slitwidth available; provide operator interaction directly (stand-alone mode); and permit interaction with other microprocessors in the measurement system (slave mode).

The controller should be extremely reliable for unattended use. In addition, the controller should also be able to operate in high noise environments without loss of accuracy and should not be the source of noise that will affect the operation of other parts of the instrument. The EU-700 (GCA/McPherson, 530 Main Street, Acton, MA, 01720, USA.) monochromator [1] was used in this study because it can be readily modified to provide these capabilities.

### Instrumentation

The monochromator controller uses both closed-loop and open-loop control systems. In both the closed-loop and the open-loop systems control signals are sent out by the microprocessor, and are modified by the control board to provide signals that can operate the electromechanical components of the monochromator. In closed-loop systems the signals originating in the monochromator are modified by the control board to provide feedback. The movements in the open-loop control systems used in this controller are assumed to be so accurate that feedback is not required.

### Wavelength drive

The wavelength drive is a sine-bar and precision leadscrew assembly driven by a slew motor for large wavelength changes (9 nm/sec) and a stepping motor for scanning and fine wavelength adjustment (zero to 2 nm/sec). The stepping motor is normally engaged. The slew motor is engaged using an ac solenoid when the slew motor is on.

### Stepping motor

The stepping motor used in the wavelength drive (No. 36D 300 R7.2, Haydon Switch and Instrument, Inc. 1502 Meriden Road, Waterbury, CN, 06705, USA.) provides reproducible wavelength increments of +/-0.01 nm/step at speeds up to 200 steps/sec. A four-phase signal is required by the stepping motor coils for forward and reverse movement. When designing the driver circuits like those in Figure 1, the designer must consider the inductive nature of the stepping motor coils. To eliminate the turn-off transients, diodes are used to shunt the current and protect the drive transistors.

### Slew motor

The slew motor (Hi-torque motor, SPEC 1218, Multi Products Company, 2052 Grove Avenue, Racine, WI, 53405, USA.) provides rapid movement of the wavelength drive between wavelengths. An alternative to the slew motor used in this monochromator design would be a high-speed stepping

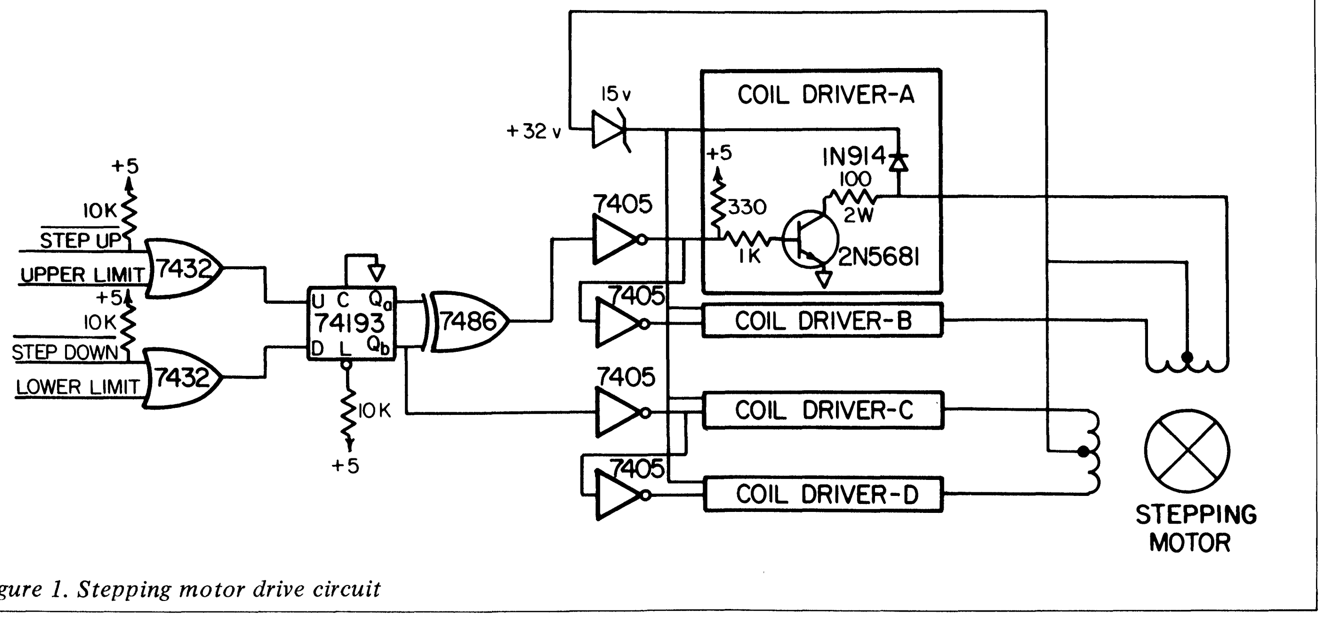

*Figure 1. Stepping motor drive circuit*

motor. This would be less attractive because high-speed stepping motors tend to be large, run hot, and require large power supplies. In general high-speed steppers are much slower (3 nm/sec max speed) than the slew motor (9 nm/sec) used in this monochromator. Major modifications to the monochromator would have been required for mounting the motor. Venting the heat produced is possible but could create problems with the optical system.

The slew motor is an ac induction motor with shading coils and a squirrel-cage rotor [2]. The application of ac line voltage to the motor and the selection of the proper pair of shading coils presents a number of design problems. If mechanical relays are used to control the slew motor power, the contacts arc, and noise spikes are created on the logic power supply lines. These noise spikes cause errors in the control logic leading to the loss of wavelength information. A solid state relay, with photoisolation and zero-crossing switching (S-312 Crydom, 1521 Grand Avenue, El Segundo, CA, 90245, USA.) is used to provide power to the slew motor, eliminating this problem.

The shading coils are connected in pairs, the direction of movement being determined by which pair of shading coils is shorted. The shading coils present a special problem because they operate at 9 volts ac peak to peak at 1.75 amps. A photoisolated solid state relay, operable at these low voltages and high current, is not presently available. However, the RCA SK3506 triac can conduct at low ac voltages and high currents, and requires low gate currents. It also provides good isolation from high voltages when off. Thus, while the monochromator controller is not completely isolated from the shading coils, the low voltages involved and the zero-crossing switching of the triac combine to prevent the creation of noise spikes. The combination of a photoisolated solid state relay on the main coil and triacs on the shading coils as shown in Figure 2 has eliminated the noise spikes and resulting errors in the wavelength counter due to slew motor operation.

### Slitwidth drive

The slits are straight knife edges adjustable from 5 to 2000 μm. The entrance and exit slits are connected to a single control shaft. A stepping motor of the same type used on the wavelength drive is directly coupled to the control shaft using a pair of miter gears.

### Filter selection

To decrease the amount of stray-light passing through the monochromator an optional filter module with a ten-position filter wheel is available. The filter wheel is positioned behind the entrance slit. The proper filter is moved into position when the filter's select line is shorted to the internal common using one of several SK3506 triacs as shown in Figure 3. The stray-light filters can be selected in three different modes with the GCA/McPherson EU-700 filter module: a manual mode, an internal automatic mode, and an external mode. The monochromator controller can select the proper filter for preselected wavelength regions stored in a table in the monochromator program when the filter module is operated in the external mode. The filter selection subroutine determines the correct filter and outputs the encoded filter number to the filter selection circuit.

### Encoder devices

In this system several encoding devices are used. These include a relative wavelength encoder, single-value absolute encoders on the wavelength and slitwidth drives, limit switches on the wavelength and slitwidth drives, and a "filter-change-in-progress" signal on the filter mechanism.

### Absolute VS relative encoders

To provide the required resolution for the wavelength drive, an absolute encoder with at least one part in ten thousand resolution (14 bits) across the wavelength range is needed. This would be prohibitively expensive.

The high cost of absolute encoders can be avoided by maintaining a wavelength counter. This counter must be set each time the instrument is turned on. A relative encoder with the proper resolution is used to update the wavelength counter. This method would preclude the complete automation of the monochromator because it normally requires the operator to initialise the counter. For this controller the relative encoder provided with the monochromator is used across the wavelength range, and an automatic absolute encoder is used for the calibration sequence at a single wavelength.

### Single-value absolute encoders

The wavelength and slitwidth absolute encoders are single value encoders. These encoders allow the automatic encoding of the wavelength to within 0.01 nm and encoding of the slitwidth to within 0.25 μm. This is as accurately as the mechanical counters on the monochromator can be manually read and set. The absolute encoders were constructed from the counters on the top of the monochromator module. The wavelength and slitwidth counters are removed from the monochromator module. The wavelength and slitwidth counters are removed from the monochromator and modified following the design of Cordos and Malmstadt [3]. The mechanical counters are then replaced in their normal position in the monochromator.

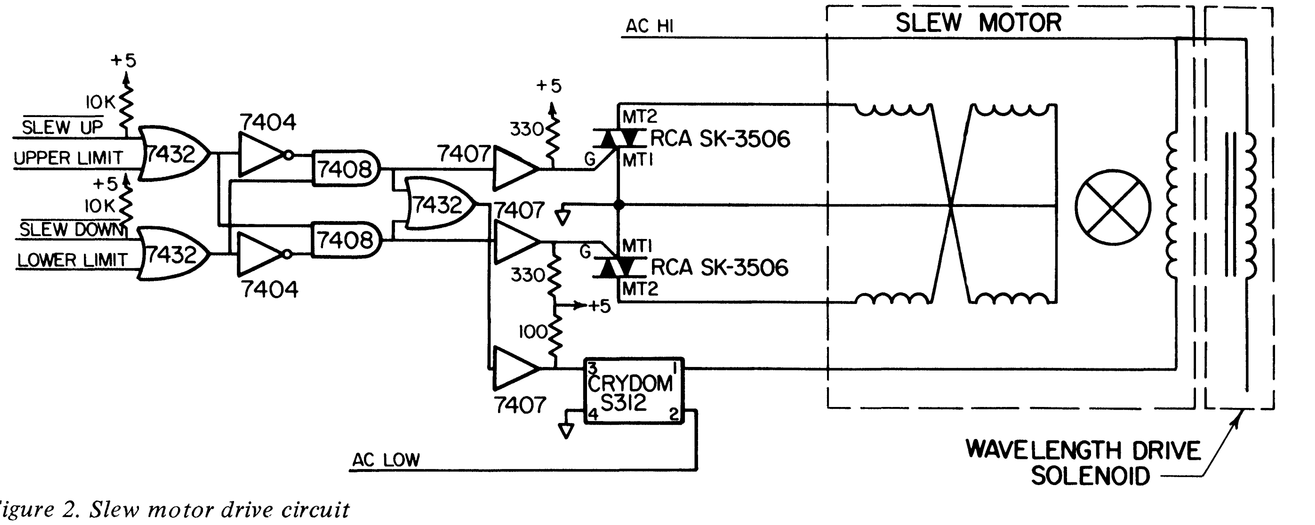

*Figure 2. Slew motor drive circuit*

The encoder as diagramed in Figures 4a, b and c, functions as follows: the incandescent lamp is turned on during calibration. At the calibration setting light can pass from the incandescent lamp to the photocell through the hole drilled through the counter rings. An interrupt transition is then generated to the microprocessor calling the proper interrupt subroutines for the wavelength and slitwidth counter. The interrupt subroutines set the wavelength and slitwidth counters for their respective encoders.

**Relative wavelength encoders**

The relative wavelength encoder is a Model B 200X AVS optical tachometer (H. H. Controls, 16 Frost Street, Arlington, MD, 02174, USA.). The optical tachometer provides quadrature output that determines the direction and magnitude of the wavelength change. The circuitry that converts the quadrature outputs to increment or decrement pulses requires the detection of an edge from each phase before producing the pulse. The circuit is shown in Figure 5. The resulting pulses generate interrupts in the microprocessor that increment or decrement the wavelength counter.

A relative wavelength encoder would not necessarily be required if movements were made only through use of the stepper motor. Because of the slew motor's inertia and the speed at which movement occurs, only large coarse wavelength changes can be made with the slew motor. Even if the slew motor were not used, however, the relative encoder would provide important assurance that the wavelength was properly set.

**Limit switches**

Another set of absolute encoders is provided by limit switches on the wavelength and slitwidth drives as shown in Figure 6a. The limit switches for both the slitwidth and the wavelength drives are used as a point of reference for the calibration procesures. The limit switches also provide important protection for the wavelength and slitwidth drives. Although the limit signals are returned and detected by the microprocessor, the limit signals are also used on the control board to prevent any movement in the direction of the limit through the board's control logic. This prevents damage to either drive.

When the sine-bar nears its limit of travel on the leadscrew, the wavelength limit switches are actuated by the limit switch actuator bar attached to the sine-bar. The slitwidth limit switches are actuated when the slitwidth stops reach their mechanical limit.

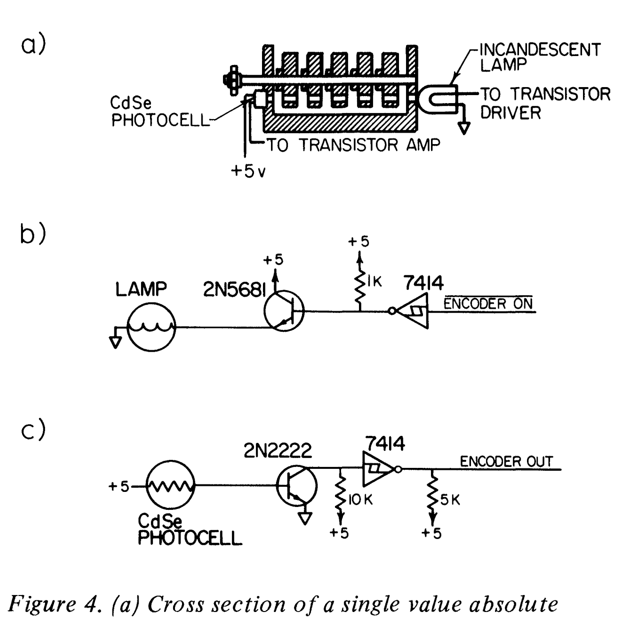

*Figure 4. (a) Cross section of a single value absolute encoder (b) Lamp drive circuit for single value absolute encoder, (c) Encoder pulse shaping circuit*

### Filter change

When the filter wheel is in a detent position, the filter-change line from the filter module is low. When a filter change is occurring, the filter-change line rises between filters. Each rising edge triggers a retriggerable monostable shown in Figure 6b, which sets the "filter-change-in-progress" signal high for one second. When the filter wheel is moving, the filter-change line pulses more than once per second. This causes the "filter-change-in-progress" signal to remain high until the filter wheel stops. The microprocessor inhibits scanning by the monochromator during the filter change interval.

### The 8080 microprocessor system

The microprocessor is the source of the control signals for the electromechanical system in the monochromator, and receives feedback signals from the monochromator, as well as instructions from the keyboard or the master microprocessor. The present status of the monochromator is

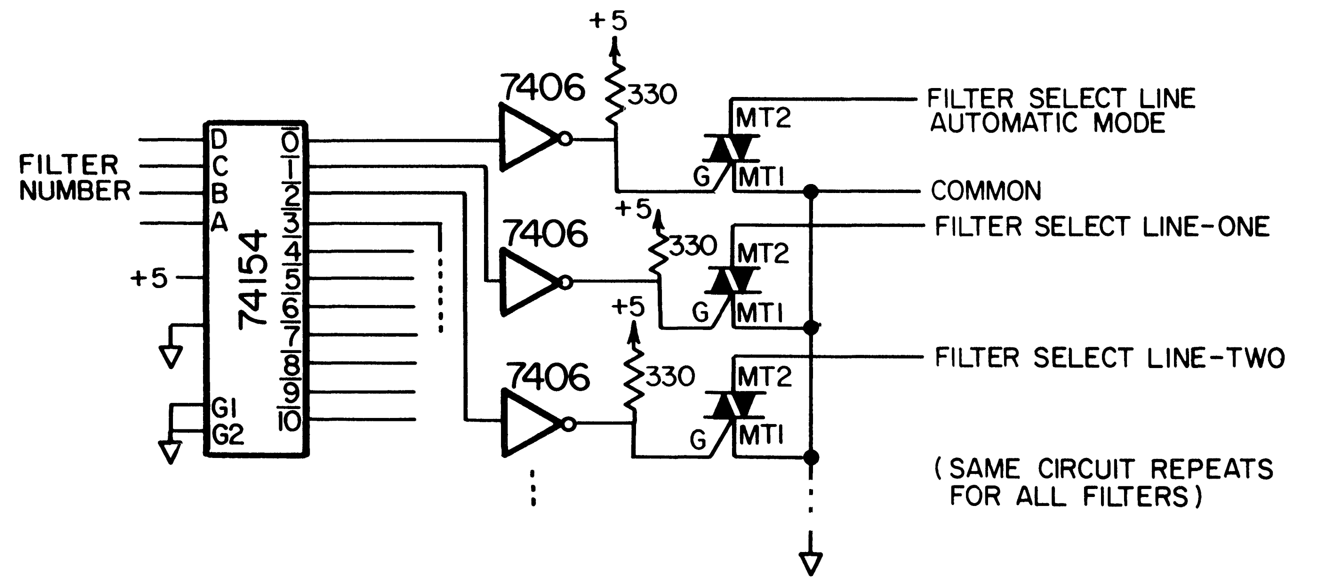

*Figure 3. Filter selection circuit*

reported either to the operator through the display or to the master microprocessor. The microprocessor in the controller is constantly testing the status of the monochromator and changing the control signals sent to the electromechanical system according to a set of internal rules determined by the monochromator control program.

The microprocessor system used in this study was designed by Avery [4] and Lovse [5], using the Intel (3065 Bowers Avenue, Santa Clara, CA, 95051, USA.) 8080A microprocessor and components in the Intel MCS-80 microcomputer family. Included are 3K bytes of read-only memory, 256 bytes of read/write memory, a keyboard and alphanumeric display, an 8251 programmable communication interface, an 8253 programmable interval timer, an 8255 programmable peripheral interface, and an 8259 programmable interrupt interface controller. These microprocessor components were interfaced using standard Intel methods. A block diagram for the microprocessor system is shown on Figure 7. The control signals passed between the microprocessor and the control board are tabulated in Table 1.

### Keyboard/display

Wavelength and slitwidth readout and stand-along control are provided by a keyboard/display unit. The display uses five DL-1414 four-digit, 17-segment alphanumeric intelligent displays (Litronix, Inc., 19000 Homestead Road, Vallcopark/ Cupertino, CA, 95014, USA.). The keyboard is composed of 20 encoded SPST keys (0-9 and A-J). The encoded signal is input through an 8212 8-bit input port by the keyboard subroutine. The encoded signal is decoded by the keyboard subroutine and the proper action is taken by the monochromator control program.

### Monochromator control program

The monochromator control program in the stand-alone mode enters commands through the keyboard. In the slave mode commands enter through a serial I/0 port which communicates with the master microprocessor. The monochromator control program can: calibrate and initialise the operation of the wavelength and slitwidth drivers, set the grating for any wavelength from 0.00 to 950.00 nm, set any slitwidth from 5 to 2000 $\mu$m, and select the proper stray-light filter. The program also detects and reports any error in the electromechanical operation of the monochromator.

The controller program is organised into an initialisation sequence, a main program loop, keyboard service subroutines, and service subroutines for the main program loop. The main loop determines if the monochromator needs service and pro-

**Table 1. Microprocessor input and output from the 8255 and 8259 interfaces.**

| | |
|---|---|
| 8255-Programmable peripheral interface | |
| Port A (Output) | |
| A0 | Wavelength slew-up |
| A1 | Wavelength slew-down |
| A2 | Wavelength step-up |
| A3 | Wavelength step-down |
| A4 | Slitwidth step-up |
| A5 | Slitwidth step-down |
| A6 | Wavelength encoder light |
| A7 | Slitwidth encoder light |
| Port B (Input) | |
| B0 | Wavelength forward limit |
| B1 | Wavelength reverse limit |
| B2 | Slitwidth forward limit |
| B3 | Slitwidth reverse limit |
| B4 | Filter-change-in-progress signal |
| B5-B7 | Not used |
| Port C (Output) | |
| C0-C3 | Filter select |
| C4-C7 | Not used |
| 8259-Programmable interrupt interface controller | |
| IR0 | Wavelength encoder up |
| IR1 | Wavelength encoder down |
| IR2 | Slitwidth calibration |
| IR3 | Wavelength calibration |
| IR4 | 200HZ clock |
| IR5-IR7 | Not used |

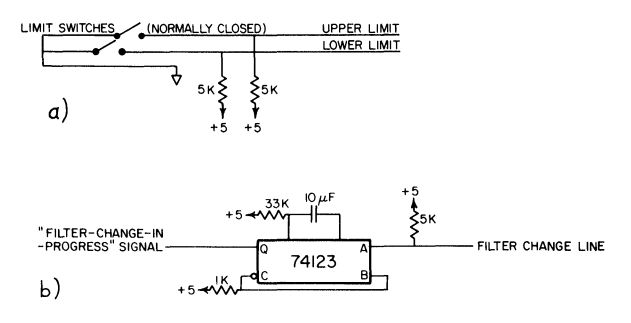

*Figure 6. (a) Limit switch pull-up circuit (b) Monostable for filter-change-in-progress signal*

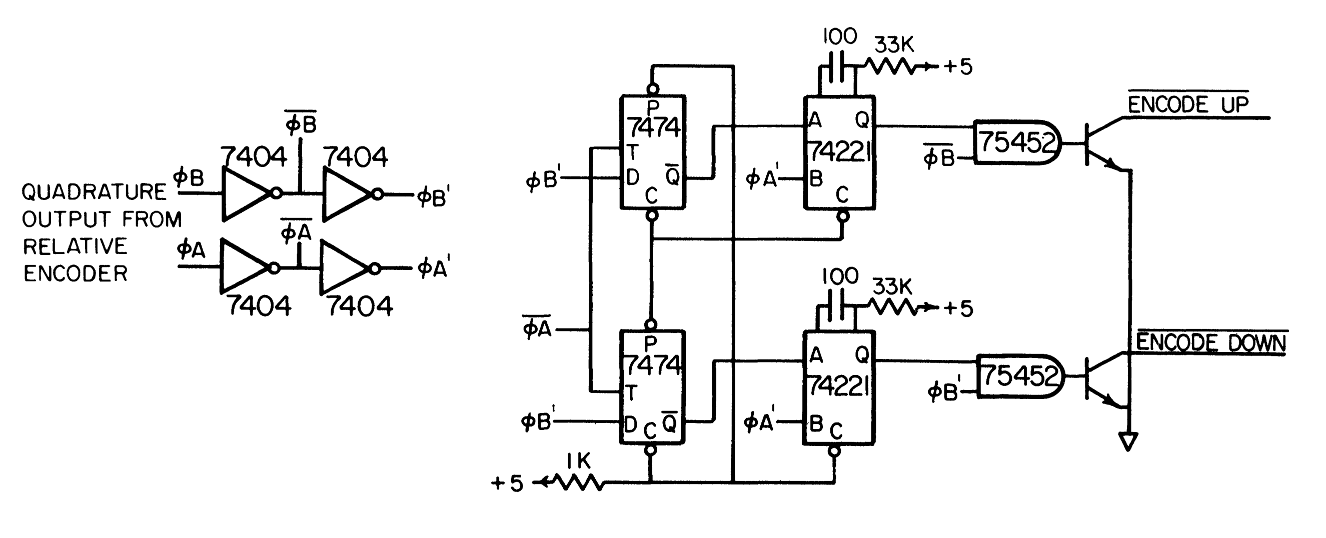

*Figure 5. Wavelength relative encoder pulse generator*

vides any needed service by executing one of the service subroutines tabulated in Table 2. Input of commands is performed by the keyboard subroutine in the stand-alone mode and by the slave subroutine in the slave mode. These subroutines then call the instruction subroutine. The instruction subroutine decodes the instruction and sets up and starts any move required. The commands and their functions are tabulated in Table 3. The program was written in assembly language because of speed requirements, since wavelength interrupts occur at 900 Hz when slew motor is on. The microprocessor must update the wavelength counter 900 times a second, and simultaneously must service the display, while continually comparing the desired and actual wavelength.

### Power supply

The control board requires 5 volts at 1 amp and 32 volts at 0.5 amps. The required voltages are supplied by an on-board power supply. The microprocessor is supplied with +5, +15, and -15 volts from a Power-One CBB-75W power supply (P.O. Box 1261, Canoga Park, CA, 19304, USA.).

### Results

The accuracy of the monochromator controller was tested with atomic lines from hollow cathode lamps. The measurement system used is shown in Figure 8. The controller was tested in both the stand-alone mode and in the slave mode. The master microprocessor used programs written in BASIC to control the slave microprocessor.

The wavelength calibration procedure was tested by first calibrating the wavelength drive, then moving to a wavelength below the 253.65 nm emission line of mercury hollow cathode lamp and stepping through the emission line. The measured emission wavelength maximum was then compared to the standard value [6]. This procedure was repeated one hundred times, and the measured peak maximum (253.55 nm) was within the accuracy of the lead screw (0.1 nm) and was reproducible to within +/- 0.01 nm.

Slitwidth calibration was tested by moving to 100, 50, and 20 μm, after calibrating the slitwidth. The intensity of light passed through the monochromator to the detector was measured 100 times at each slitwidth. The measured intensity never varied more than one per cent for any slitwidth setting. This corresponds to an error of 0.2 μm in the slitwidth setting.

Accuracy during unattended operation was tested for as long as 8 hours while the monochromator controller was in the slave mode. The monochromator wavelength drive was in continuous movement during the test period. At the end of the test period the monochromator controller wavelength error was less than 0.01 nm. This is within the resolution of the monochromator. The error for the slitwidth movement was less than the step resolution or less than 0.25 μm.

Because of the high precision of this controller the wavelength accuracy error caused by the leadscrew run-out error for a portion of the wavelength range could be measured. The measured peak maximums were compared to the known wavelengths for specific lines, and the error was calculated as shown in Table 4. Although some of the measured errors are

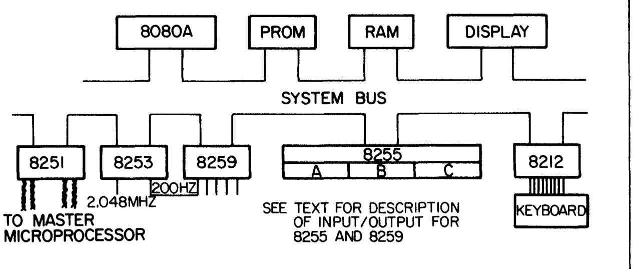

*Figure 7. Microprocessor system*

**Table 2. Main program loop service subroutines**

| | |
|---|---|
| KEYBD | Determines if a valid input has occured from the key - board and takes any required action when enabled. |
| SLAVE | Determines if a valid input has occured from the slave input port when in the slave mode. |
| SMOVIN | Supervises any change in the slitwidth. |
| MOVING | Supervises any change in the wavelength. |
| DISPLA | Displays the current values for the slitwidth and the wavelength. |
| MCSLIT | Supervises the slitwidth calibration procedure. |
| MCWAV | Supervises the wavelength calibration procedure. |
| ERROR | Determines if an error in electromechanical operation has occured. |
| FILTER | If enabled selects the proper stray-light filter. |

**Table 3. Microprocessor commands**

| | |
|---|---|
| COPY (B) | Sets wavelength counter equal to value in the input buffer |
| CSLIT (B) | Starts slitwidth calibration procedure |
| CWAVE (B) | Starts wavelength calibration procedure |
| DISLW (B) | Disables wavelength slew |
| ENSLW (B) | Enables wavelength slew |
| FILDIS (B) | Diables filter selection |
| FILENA (B) | Enables filter selection |
| FILSEL (M) | Uses filter number stored in the input buffer to select the correct filter |
| GOTO (B) | Starts wavelength change to wavelength in the input buffer |
| OUTFIL (M) | Outputs the current filter number to the master microprocessor |
| OUTSLI (M) | Outputs the current slitwidth to the master microprocessor |
| OUTWAV (M) | Outputs the current wavelength to the master microprocessor |
| SCOPY (B) | Sets slitwidth equal to value in the input buffer |
| SGOTO (B) | Starts slitwidth change to value stored in the input buffer |
| SHOFF (B) | Turns keyboard shift off |
| SHON (B) | Turns keyboard shift on |
| SLAVOF (M) | Returns microprocessor to the stand-alone mode |
| SLAVON (M) | Places microprocessor in slave mode |
| STATUS (M) | Outputs current status to the master microprocessor |
| SSTEPD (B) | Steps the slitwidth down 0.25 μm |
| SSTEPU (B) | Steps the slitwidth up 0.25 μm |
| STEPDN (B) | Steps the wavelength drive down 0.01 nm |
| STEDUP (B) | Steps the wavelength drive up 0.01 nm |
| STOP (B) | Stops any monochromator movement |
| WAITW (B) | Sets wavelength scanning speed using value in the input buffer |
| WAITS (B) | Sets slitwidth scanning speed using value in the input buffer |

B – Commands available to both the keyboard and the master mircroprocessor

M – Commands available only to the master microprocessor

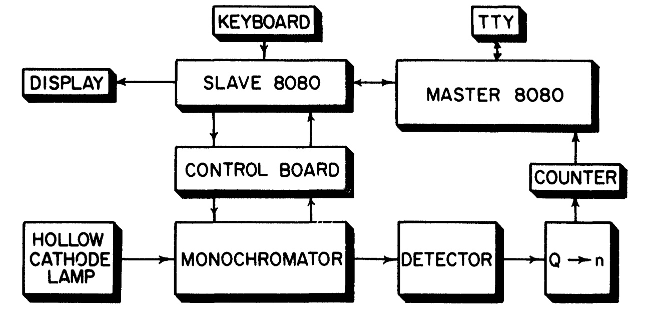

*Figure 8. Experimental measurement system*

greater than the monochromator's specification, it should be noted that the monochromator used in this study is over ten years old. Because this error is a rather smooth and stable function of wavelength, it is possible to calculate the wavelength of an unknown emission line in a complex sample to an accuracy better than the monochromator specification. The measured wavelength is slightly dependent on temperature, as shown in Table 4. Thus, for the best accuracy, the monochromator should be calibrated at the temperature at which it is to be used. Also, data can be collected for a few points in the region of interest, and the wavelength shift can be calculated for the operating temperature by comparing these data to the calibration values at a standard temperature. The microprocessor controller enables the overall performance of the monochromator to be improved beyond its basic specifications, as well as providing the automation features.

*Software for the control programs, artwork for the PC boards and complete documentation are available from the authors.*

**Table 4. Wavelength accuracy reproducibility and temperature data for the EU-700 monochromator**

| Temperature (°C) | Average value | Literature value (6) | Error nm |
|---|---|---|---|
| 24.0 | 253.54 | 253.65 | −0.11 |
| | 365.06 | 365.01 | +0.05 |
| | 404.73 | 404.66 | +0.07 |
| | 435.90 | 435.84 | +0.06 |
| | 546.10 | 546.08 | +0.02 |
| 25.1 | 253.55 | 253.65 | −0.10 |
| | 365.09 | 365.01 | +0.08 |
| | 404.77 | 404.66 | +0.11 |
| | 435.91 | 435.84 | +0.07 |
| | 546.12 | 546.08 | +0.04 |
| 27.1 | 253.57 | 253.65 | −0.08 |
| | 365.10 | 365.01 | +0.09 |
| | 404.77 | 404.66 | +0.11 |
| | 435.92 | 435.84 | +0.08 |
| | 546.13 | 546.08 | +0.05 |

REFERENCES

[1] GCA Corporation, "Scanning Monochromator EU-700 and EUE-700 Series", Acton, MA, USA, 1968.

[2] Fractional and Subfractional Horsepower Electric Motors. C.G. Veinott, McGraw-Hill Book Company, New York, 1970.

[3] Cordos, E., and Malmstadt, H.V., (1975) *Anal. Chem.* **45**, 425(2), "Programmable Monochromator for Accurate High Speed Wavelength Isolation".

[4] Avery, J., Ph D. Thesis, University of Illinois, School of Chemical Sciences, Urbana, IL, 1978.

[5] Lovse, D., Ph D. Thesis, University of Illinois, School of Chemical Sciences, Urbana, IL, 1977.

[6] The Chemical Rubber Company, Handbook of Chemistry and Physics, Cleveland, OH, 1970, p E-220.

### Novel apparatus for the automation of solvent extraction

**John G. Williams, Peter B. Stockwell*, Michael Holmes, Derrick G. Porter.**

*Laboratory of the Government Chemist, Stamford St., London SE1 9NQ, UK.*

### Introduction

Chemical analyses usually require pretreatment of the sample prior to measurement. When solutions are employed as transport media for the analytes, convenient minimisation of physical and chemical interferences may be brought about by solvent extraction. Previously, automatic solvent extraction systems have been based on manual techniques, whereby the eye of the human operator is replaced by some form of phase boundary detector and the tap of the separating funnel is replaced by an electromechanical valve. Most phase boundary sensors have broadly similar characteristics. Apart from operating problems they all rely on the differential transmission of electromagnetic radiation on either side of the boundary. Trowell has described phase boundary detectors based on differential conductivity [1] and differential capacitance [2]. Associated with the latter, relying on a dielectric change, are methods using a change of refractive index [3] to control a bistable valve as an interface flows past a fixed point in the system. Recent, unpublished work performed at this Laboratory has shown that ultrasonic transducers are also capable of phase boundary detection.

Other well tried forms of solvent extraction (other than chromatographic) involve the migration of the species of interest across a semi-permeable membrane under the influence of either a concentration gradient or a potential gradient, or a combination of the two. Methods relying on the gravity separation of two completely immiscible phases are sometimes employed in continuous-flow air-segmented analytical systems; when well designed they are relatively trouble free. Vallis [4] designed a somewhat different approach to automated solvent extraction based on a rotatable cup-shaped vessel with a porous lid attached to the lip. The cup is placed inside a collecting vessel; if the porous lid is made from hydrophilic material such as sintered glass, water will pass into the collecting vessel at low rotation speeds leaving the organic phase in the cup. An increased rotation speed then ejects the organic phase. The use of a hydrophobic material such as sintered PTFE enables the preferential rejection of the organic phase.

A prototype separator which has been designed and built at this Laboratory using a completely new approach, is currently the subject of a patent application [5]. In principle, separation is effected by absorption of both phases into a porous nickel-chrome alloy disc mounted on a motor-driven shaft. Controlled angular acceleration and centripetal force on the droplets within the pores enables one phase to be separated from the other. The speed of rotation of the porous disc is coupled microelectronically to the vertical component of its motion so that separated droplets leaving the disc tangentially are trapped by hitting the walls of

*Present address: *Plasma Therm Ltd., 6 Station Road, Penge, UK.*

